# Scaling up and institutionalising multisectoral collaboration and policy action for non-communicable diseases and mental health in South Africa: Lessons, gaps and insights from the 2025 Diabetes Summit

**DOI:** 10.4102/phcfm.v18i2.5573

**Published:** 2026-07-30

**Authors:** Kibachio J. Mwangi, Patrick Ngassa Piote, Melanie Pienaar, Masindi Mashapha, Nkateko Mkhondo, Mikkel Pape Dysted

**Affiliations:** 1 World Health Organization, Pretoria, South Africa; 2 School of Health Systems and Public Health, Faculty of Health Sciences, University of Pretoria, Pretoria, South Africa; 3 Department of Public Health, Medical School, Nelson Mandela University, Gqeberha, South Africa; 4 School of Nursing, Faculty of Health Sciences, University of the Free State, Bloemfontein, South Africa; 5 Department of Social Development, Pretoria, South Africa; 6 World Diabetes Foundation, Copenhagen, Denmark

## Introduction

The 2025 South African Diabetes Summit, convened by the Diabetes Alliance, the World Health Organization (WHO) and the National Department of Health (NDoH), brought together policymakers, clinicians, researchers, civil society and people living with diabetes to reflect on the growing burden of diabetes and other non-communicable diseases (NCDs) in South Africa.

The Summit positioned diabetes as a tracer for the broader NCD and mental health crisis and stressed the urgency of coordinated, multisectoral responses.

As many NCD drivers lie outside the health sector, effective responses require coherent policies across finance, trade, social affairs, economic development and education within a whole-of-government approach.^1^

South Africa’s NCD National Strategic Plan 2022–2027 places NCDs+ (including mental health and neurological conditions) at the centre of Universal Health Coverage (UHC), aligned to the Sustainable Development Goals (SDGs) and Health-in-All-Policies and advances a 90%:60%:50% cascade for detection, treatment and control.^2^

These priorities align with the WHO and the United Nations Inter-Agency Task Force (UNIATF) on NCDs, which emphasise policy action beyond the health sector and call for stronger governance, accountability and shared capacity, particularly in addressing structural drivers such as poverty, inequality, unemployment, food insecurity and low health literacy that continue to shape NCD risk and outcomes.^3^

Drawing on Summit deliberations, this article: (1) outlines South Africa’s multisectoral landscape for NCDs and mental health, (2) identifies barriers to routine collaboration and (3) proposes a feasible agenda to scale and institutionalise multisectoral action for measurable population and equity gains.

## Why multisectoral action is essential for non-communicable diseases and mental health

Non-communicable diseases and mental health conditions are largely shaped by determinants outside the health sector. Food and alcohol environments, tobacco marketing, housing and transport, education and employment, social protection, safety and commercial practices all influence risk exposure, service access and continuity of care.^4^ In South Africa, these drivers intersect with inequality and a high burden of communicable diseases, creating multimorbidity and complex needs at the primary health care (PHC) level.

Diabetes remains a leading cause of death, reflecting a shift to NCD-dominated mortality. Discussions on multisectoral collaboration highlighted how social, economic and environmental determinants drive NCDs and identified opportunities to strengthen prevention, early detection and long-term care.

Participants noted pockets of strong policy and programme action but highlighted uneven implementation, limited measurement of prevention impact, fragmented coordination and enforcement and weak community engagement as key barriers to scaling up and reducing premature mortality and costs.

Participants highlighted poverty, unemployment, inequality and food insecurity as key structural drivers of ill-health, shaping behavioural risks such as poor diet and tobacco use, as well as access to prevention and care. Concerns were raised about the growing availability of processed foods and limited consumer awareness of their risks, with these commercial and social determinants contributing to the rising diabetes burden.

Discussions also underscored the intersection between NCDs and communicable diseases, particularly in settings of poverty and overcrowding, leading to complex multimorbidity that must be managed within PHC. Multisectoral action was therefore framed not as optional, but as a core governance approach for aligning policies, services and resources across sectors.

Effective responses require alignment of policies, budgets, regulations, services and community participation to address social, environmental and commercial determinants while strengthening integrated chronic care. This includes policy packages to reduce population risk exposure and service reforms to improve detection and control, especially among underserved groups.

World Health Organization guidance calls for Health-in-All-Policies and whole-of-government and whole-of-society approaches to create health-promoting environments and enable prevention and equitable care.^4,5^

## South Africa’s multisectoral landscape

The National Strategic Plan for the Prevention and Control of NCDs 2022–2027 and the National Mental Health Policy Framework and Strategic Plan 2023–2030 emphasise stronger governance, partnerships and accountability and position PHC and district health systems as the foundation for integrated chronic care.^2,6^

The 2025 Diabetes Summit itself reflected a whole-of-society approach, bringing together diverse stakeholders to align priorities and promote shared learning, with participants noting the value of such platforms for collaboration and accountability. While examples of multisectoral policy measures discussed, for instance, fiscal interventions such as the Health Promotion Levy and regulatory actions targeting tobacco and the food environment,^7,8,9^ showed that multisectoral action is underway, it was noted to be often fragmented.

The Department of Social Development highlighted its role in addressing structural drivers through social protection, counselling and community outreach. Participants noted that diabetes and other NCDs share determinants such as poverty, food insecurity and barriers to care with tuberculosis (TB). The TB response provided useful lessons on multisectoral collaboration, community engagement and shared accountability for addressing these drivers.

Despite these efforts, the Summit concluded that there is no adequately coordinated mechanism for multisectoral action on diabetes and other NCDs, and that there are major gaps in prevention measurement, surveillance, care and support. Fragmented responsibilities and uneven implementation across levels of government hinder alignment of policies, budgets and delivery. Policy cohesion remains ad hoc, and reliance on vertical programmes is unsustainable, underscoring the need for stronger coherence and accountability across national, provincial and district levels.

## Barriers to effective multisectoral collaboration

World Health Organization’s 2025 thematic issue briefs identify four levers for sustained NCD multisectoral collaboration: (1) governance and accountability, (2) leadership at all levels, (3) ways of working and (4) resources and capabilities.^1^

This session on institutionalising multisectoral collaboration and policy action for NCDs and mental health in South Africa highlighted several interrelated barriers across these levers that continue to hinder multisectoral action on NCDs.

Firstly, structural challenges such as poverty, inequality and unemployment were identified as fundamental drivers of poor health outcomes. These factors not only increase vulnerability to NCDs but also limit access to care and the effectiveness of interventions.

Secondly, participants emphasised persistent gaps in public awareness and health literacy, particularly regarding nutrition, diet and the commercial determinants of health. This limits the effectiveness of prevention efforts and contributes to ongoing risk exposure.

Thirdly, stigma and gender-related barriers were highlighted as important constraints to care. Participants noted that stigma can limit disclosure and care-seeking, particularly among men, and emphasised the need for community-based interventions to address these challenges.

Fourthly, fragmentation across sectors and weak coordination mechanisms were identified as major systemic barriers. Participants highlighted the absence of integrated reporting systems and limited alignment across government departments, which undermines implementation and accountability.

Fifthly, limitations in surveillance and data systems were noted as critical challenges. Weak data integration and limited capacity to measure prevention outcomes constrain the ability to monitor progress and guide decision-making.

## Opportunities for strengthening multisectoral collaboration

Building on the Summit discussions, four practical opportunities for strengthening multisectoral collaboration were highlighted: (1) establishing joint monitoring and accountability systems with harmonised indicators, integrated data sharing; and real-time reporting; (2) expanding collaborative research and innovation on social, economic, commercial and environmental determinants to co-develop context-appropriate interventions; (3) strengthening capacity building and cross-provincial and district learning exchanges; and (4) mobilising sustainable financing by engaging government and non-government stakeholders to support cost-effective interventions.

The 2025 Diabetes Summit emphasised that stronger collaboration must address upstream determinants and build practical delivery partnerships. The social development sector underscored the roles of poverty, inequality, gender barriers and stigma, especially among men, and the importance of social protection and food security.

Lessons from the TB programme highlighted the need to address determinants, promote shared accountability and leverage established structures such as the South African National Aids Council (SANAC) as transferable models. Partners and civil society stressed prioritisation under fiscal constraints, meaningful engagement with non-health sectors and transparent, people-centred collaboration.

The Summit also promoted innovations to make action routine, including embedding social determinants in programme design, using community outreach for stigma reduction and health promotion, scaling through cross-sector learning platforms and strengthening data-driven accountability with shared indicators and routine reporting.

The role of civil society and community engagement was strongly emphasised, including people-centred communication, inclusive engagement and safe spaces for dialogue to address stigma and raise awareness.

Participants noted that institutionalisation requires a clear, feasible package that defines roles, timelines and measures of success.^8^ In South Africa, multisectoral mapping should specify what each department delivers, how actions are financed and how progress is tracked, recognising that key drivers lie beyond the health system. They also suggested drawing on existing international frameworks aligned to Millennium Development Goals (MDGs) and SDGs commitments to guide action on NCDs.

## Conclusion and way forward

The 2025 Diabetes Summit demonstrated that while South Africa has a strong policy foundation for NCDs and mental health, progress is constrained less by a lack of policies than by weak institutionalisation of multisectoral action, fragmented accountability and insufficient measurement of prevention and population-level impact.

A key contribution of the Summit was the identification of practical opportunities to move multisectoral collaboration from ad hoc engagement to routine, accountable implementation.

To accelerate progress, the NDoH and partner departments should establish a formal multisectoral coordination and accountability mechanism for NCDs and mental health, with clear roles, shared indicators and integrated monitoring systems. Provincial and local governments should operationalise these commitments through district and municipal compacts linked to District Development Plans.

The Departments of Social Development, Basic Education, Agriculture, Trade and Transport should strengthen action on the social, environmental and commercial determinants of health, while research institutions, civil society, and development partners support surveillance, community engagement, innovation and accountability.

Regular national platforms such as the Diabetes Summit should serve as accountability forums to review progress, share learning, mobilise partners and sustain momentum towards improved population health and equity.
